# Myotonometry and Muscle Force in Patients with Surgically Treated Tibial Pilon Fracture: A Cross-Sectional Study

**DOI:** 10.3390/jfmk11010021

**Published:** 2025-12-31

**Authors:** Andrei-Daniel Bolovan, Gheorghe-Bogdan Hogea, Elena-Constanta Amaricai, Alexandra-Roxana Tapardea, Ahmed Abu-Awwad, Liliana Catan

**Affiliations:** 1 Doctoral School, “Victor Babes” University of Medicine and Pharmacy, 300041 Timisoara, Romania; andrei.bolovan@umft.ro (A.-D.B.); roxana.tapardea@umft.ro (A.-R.T.); 2 Research Center for Assessment of Human Motion, Functionality and Disability, Department of Rehabilitation, Physical Medicine and Rheumatology, “Victor Babes” University of Medicine and Pharmacy, 300041 Timisoara, Romania; catan.liliana@umft.ro; 3 Department of Orthopedics and Traumatology, “Victor Babes” University of Medicine and Pharmacy, 300041 Timisoara, Romania; hogea.bogdan@umft.ro (G.-B.H.); ahm.abuawwad@umft.ro (A.A.-A.); 4 “Pius Brinzeu” Emergency Clinical County Hospital, Bld. Liviu Rebreanu, No. 156, 300723 Timisoara, Romania; 5 Research Center Teodor Sora, Department of Orthopedics II, “Victor Babes” University of Medicine and Pharmacy, Eftimie Murgu Square, No. 2, 300041 Timisoara, Romania

**Keywords:** tibial pilon fracture, myotonometry, stiffness, muscle strength, dynamometry

## Abstract

**Background:** Tibial pilon fractures are, in most cases, complex injuries caused by high-energy trauma. This type of fracture requires surgical stabilization and immobilization that impairs ankle function by reducing range of motion, muscle strength, and affecting the mechanical properties of the muscles. **Methods:** We evaluated 22 patients who required surgery for tibial pilon fractures and 22 age-matched healthy controls. Dynamometry assessed the isometric strength of the dorsiflexors and plantar flexors. Myotonometry of the tibialis anterior, peroneus longus, and medial and lateral gastrocnemius muscles analyzed the muscle tone, biomechanical (stiffness and decrement), and viscoelastic properties (mechanical stress relaxation and ratio of relaxation time to deformation time (creep). **Results:** Compared to the control group, the patients had significantly decreased isometric strength in both the dorsal flexors and plantar flexors on the affected side. Myotonometric measurements did not reveal significant differences in the tibialis anterior and peroneus longus muscles. Both medial and lateral gastrocnemius muscles exhibited significantly increased frequency and stiffness, and significantly decreased relaxation and creep in patients when compared to the control group. **Conclusions:** When compared to healthy controls, patients with surgically treated unilateral pilon fracture had a decreased isometric muscle force of ankle dorsiflexors and plantar flexors of both affected and non-affected lower limbs. Myotonometry indicated increased frequency and stiffness, along with decreased values of viscoelastic parameters (stress relaxation time and creep) in the medial and lateral gastrocnemius muscles on both sides.

## 1. Introduction

Fractures of the tibial pilon are typically caused by axial loading combined with rotational forces. Treatment is often challenging due to the frequent association with high-energy injury patterns and soft-tissue damage, coupled with the limited soft-tissue envelope at the fracture site [1,2]. This type of fracture commonly requires surgery and prolonged immobilization. Although surgery addresses bone realignment, long-term immobilization and weight-bearing restrictions cause muscle atrophy and weakness, as well as a restricted range of motion (ROM). The ankle’s anatomy, with its limited soft-tissue coverage, increases the risk of scarring, adhesions, and poor healing after trauma or surgery [3].

While extensive research has focused on osseous healing and radiographic outcomes after pilon fractures [4,5,6], there is insufficient understanding of how muscles adapt after these fractures and how their intrinsic properties change. Periarticular muscles (gastrocnemius, tibialis anterior, and peroneus longus) play key roles in providing dynamic ankle stability, enabling an efficient gait pattern and balance control [4,5].

After a pilon fracture and immobilization, the muscles mentioned above are susceptible to disuse atrophy, altered neuromuscular activation, and structural changes within their extracellular matrix. Additionally, scar tissue and fibrosis can disrupt the normal viscoelastic properties by causing abnormal collagen deposition, altered cross-linking, changes in proteoglycan content, and impaired water balance [6,7,8]. As a result, these changes can increase muscle stiffness, reduce elasticity, and impair the muscle’s ability to absorb and transmit forces during movement. Some patients could fail to regain full muscle strength after surgery due to muscular inhibition that causes beyond normal functional mobility. Adequate strength is needed in the calf to maintain proper gait mechanics during everyday activities [9].

Impaired performance of the dorsiflexor and plantar flexor muscles, along with limited range of motion, has significant consequences for gait biomechanics. Weakness in push-off power, ankle stability, and proprioception extends the rehabilitation process and limits the return to daily and recreational activities [10]. Houben et al.’s [1] study indicated that patients with pilon fractures exhibit reduced flexion/extension and inversion/eversion at the hindfoot-tibia interface, likely at the talocrural joint, and compensate by increasing abduction/adduction in nearby joints in this area. The correlation between adduction/abduction and functional outcomes suggests that greater compensatory motion in the unaffected joints is associated with better patient-reported results. Abnormal gait patterns are often observed in patients after pilon fracture fixation, reflecting not only joint impairment but also underlying changes in muscle and soft tissue [1].

Myotonometry, a non-invasive method, assesses muscle tone and biomechanical and viscoelastic properties (stiffness, elasticity, relaxation time, and creep) [11]. Studies have shown that it detects changes in ankle muscles after lateral ankle sprains and in individuals with chronic ankle instability (CAI). Subjects with a history of sprains or CAI often exhibit increased muscle tone and stiffness, along with reduced elasticity in the tibialis anterior and peroneus longus muscles [12,13]. Still, there is limited understanding of how surgically treated pilon fractures affect the biomechanical and viscoelastic properties of muscles and how these changes might lead to functional impairments.

The objectives of the current study were to assess ankle muscles in patients with surgically treated pilon fractures and to compare these data with those of healthy subjects. The evaluations consisted of myotonometry (muscle assessment in a relaxed state) and dynamometry (isometric voluntary contraction). We hypothesized that, in addition to reduced ankle ROM and strength, patients would exhibit changes in myotonometric parameters of the examined muscles, characterized by increased muscle tone and stiffness, along with reduced elasticity, relaxation time, and creep, compared with the control group.

## 2. Materials and Methods

### 2.1. Study Design

**Figure 1:**
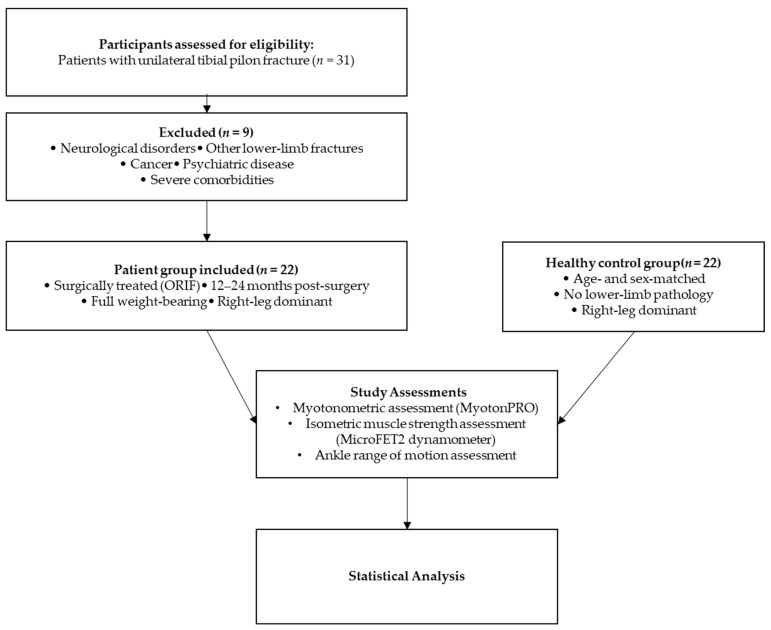
Flow diagram of the study design and assessment protocol.

This study was designed as a cross-sectional study comparing the myotonometer parameters and isometric muscle force of the ankle muscles in patients who had suffered a unilateral tibial pilon fracture and gender and age-matched healthy volunteers (Figure 1).

### 2.2. Participants

A total of 31 patients with surgically repaired unilateral tibial pilon fractures participated in the study (Figure 1). The period from surgery to patient evaluation varied from 12 to 24 months, allowing for complete fracture healing and functional recovery. Inclusion criteria required clinical and radiological evidence of fracture healing, the ability to bear full weight on the affected limb, and the affected limb being the dominant one. Exclusion criteria consisted of the following: history of trauma or bone fractures in either the affected or opposite limb, neurological or health conditions that impair walking or muscle function, and leg length discrepancy not associated with the tibial pilon fracture. Patients with psychiatric disorders, severe cardiovascular disease, morbid obesity (BMI > 40), or cancer were also excluded due to potential risks affecting compliance and follow-up.

All patients included in the study underwent open reduction and internal fixation (ORIF). In cases with extensive soft-tissue injury or swelling, temporary external fixation was initially used, followed by definitive ORIF once local conditions permitted, usually within 7–14 days. Fixation was performed with anterior or anterolateral locking plates and screws under fluoroscopic guidance.

Nine patients were excluded due to specific conditions: three with lumbar disk hernia, two with stroke, one with hip fracture, one with calcaneus fracture, one with cancer, and one with depression. Additionally, healthy volunteers matched for gender and age were recruited as controls. Participation was voluntary, and all participants provided written informed consent. The research was approved by the Ethics Committee of “Victor Babes” University of Medicine and Pharmacy in Timisoara, Romania (reference no. 26/2023-08-25), and complied with the Helsinki Declaration. Clinical data, including age, height, weight, and body mass index, were collected from both patients and controls. Both patients and controls exhibited right-leg dominance.

The study included 22 patients with surgically treated tibial pilon fractures and 22 age-matched healthy controls. The patients and control groups were homogenous, as we found no statistically significant differences in their characteristics (Table 1).

### 2.3. Assessment

**Figure 2:**
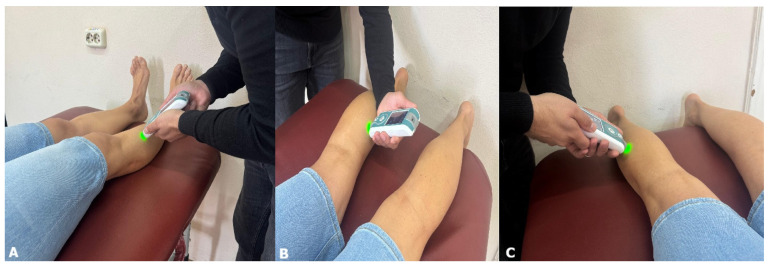
Myotonometric assessment points and participant positions for the tibialis anterior (**A**), medial gastrocnemius (**B**), and lateral gastrocnemius (**C**) muscles.

For the myotonometric assessment (Figure 2), we used the Myoton PRO Digital Palpation Device (Myoton AS, Tallinn, Estonia) with software version 5.0.0.232 [11]. We assessed the tibialis anterior in the anterior compartment, the gastrocnemius in the posterior compartment, and the peroneus longus in the lateral compartment. For the assessment of the tibialis anterior and peroneus longus, participants lay in a supine position. For the medial and lateral gastrocnemius, measurements were conducted in the prone position. Participants had to rest in these positions for at least 1 min before the tests, with all assessments performed in a relaxed position. The probe of the device was positioned perpendicularly to the skin over the thickest part of each muscle belly. For the tibialis anterior, the measurement point was located at the proximal third of the distance from the tibial tuberosity to the fibular malleolus. For the peroneus longus, the probe was applied at the proximal third of the distance between the fibular head and the fibular malleolus. For the gastrocnemius, the medial and lateral heads were assessed at the midpoint between the popliteal fossa and the calcaneal insertion. These anatomical landmarks have been used in previous studies to ensure consistency and reproducibility of Myoton measurements [12,13,14]. The myometer applies a sequence of 5 mechanical impulses (1 s apart), with a pre-load of 0.18 N, and then further compresses subcutaneous tissue with an additional 15 ms impulse of 0.40 N of mechanical force to the central part of the muscles. The test was repeated if the coefficient of variation in the mechanical impulses exceeded 3%. The following parameters were analyzed: frequency (Hz), stiffness (N/m), decrement, relaxation time (ms), ratio of relaxation time to deformation time (creep) [11].

**Figure 3:**
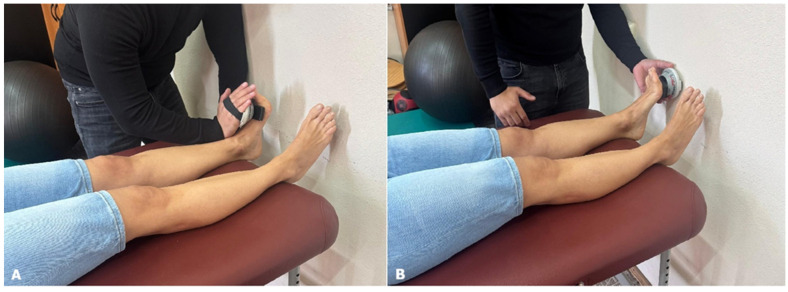
Isometric strength testing using the handheld dynamometer (MicroFET2) during ankle dorsiflexion (**A**) and plantarflexion (**B**).

Isometric muscle force was assessed using the handheld MicroFET2 dynamometer from Hoggan Scientific [15], a portable, precise device designed to obtain objective, reliable, and quantifiable muscle-testing measurements [16,17]. The device records force in newtons (N), reflecting the isometric muscle force generated during contraction. Participants were barefoot and performed ankle plantarflexion and dorsiflexion while lying supine with their hips and knees extended and their ankles in a neutral position. The dynamometer was placed over the metatarsal heads on the sole for plantar flexion, and on the top of the foot for dorsiflexion (Figure 3), with the examiner applying steady resistance during 3 s contractions [16,18]. Both muscle tests were repeated three times, with 5 s of rest between trials. The analysis considers the mean of the three trials. Muscle strength was evaluated in both affected and unaffected lower extremities.

The range of motion of both right and left ankles was recorded (dorsiflexion, plantar flexion, inversion, and eversion) (Table 2). The range of ankle motion was assessed with a standard manual goniometer. On the right side, range of motion was significantly decreased across all movements in the patient group compared with controls.

All the tests were performed at a constant room temperature (21–23 °C). The same trained orthopedic doctor (A.-D.B.) applied myotonometry, dynamometry, and goniometry.

In order to evaluate the intra-rater reliability of the myotonometer and of the handheld MicroFET2 dynamometer, the investigator (A.-D.B.) performed the tests in 10 healthy controls after a 4-week interval between the first and second assessments. For the intra-rater reliability, we analyzed the measurement data of the right dominant lower limb.

### 2.4. Statistical Analysis

Data analysis was performed using GraphPad Prism version 5.0. Descriptive statistics, such as mean and standard deviation, were computed for all variables. Before statistical analysis, the normality of the values in this study was verified using the D’Agostino-Pearson normality test. Differences in myotonometric parameters and muscle force between patients and controls were evaluated using Student’s unpaired *t*-test or a Chi-squared test. Effect sizes for comparisons between patients with surgically treated tibial pilon fractures and healthy control groups were assessed using Cohen’s *d* (effect size), with Cohen’s *d* > 0.8 considered a large effect size [19]. Results were considered statistically significant when the *p*-value was below 0.05 [20].

## 3. Results

The isometric force of the tested muscles (ankle dorsiflexors and ankle plantar flexors) in patients and controls is presented in Table 3. When comparing the right ankle dorsiflexors, we observed a statistically significant decrease in muscle force on the affected side in patients; isometric muscle force on the affected side was lower than on the dominant side in healthy subjects. The same findings were recorded for the right ankle plantar flexors. When comparing the left ankle dorsiflexors and left ankle plantar flexors (non-injured side in patients and non-dominant side in healthy controls), no significant differences were noted between patients and controls.

Table 4 and Table 5 present comparisons of myotonometric parameters for the anterior tibialis and longus peroneus muscles, respectively. When we compared the right sides of patients and controls, no significant differences were observed in any of the five parameters (frequency, stiffness, decrement, relaxation, and creep) for the anterior tibialis and longus peroneus muscles, respectively. The same findings were recorded for the left side.

The myotonometric parameters of the medial gastrocnemius muscle in patients and controls are presented in Table 6. When comparing the right side (the injured side for patients and the dominant side for controls), significant differences were noted for all the assessed parameters. When comparing the left side (the non-affected side for patients and the non-dominant side for controls), significant differences were also observed for the five parameters. In the patient group, frequency (state of tension) and stiffness were significantly increased, while elasticity (decrement) and viscoelastic parameters (relaxation and creep) were significantly reduced on both the affected and non-affected sides.

The myotonometric parameters of the lateral gastrocnemius muscle in patients and controls are presented in Table 7. When comparing the right side (injured side for patients and dominant side for controls) and the left side (non-injured side for patients and non-dominant side for controls), significant differences were observed in frequency, stiffness, relaxation, and creep. Frequency (state of tension) and stiffness were higher in the patient group, whereas the viscoelastic parameters (relaxation and creep) were significantly lower.

The intra-rater reliability was expressed as ICC (intra-class correlation coefficient). Considering the myotonometry, for frequency parameter we recorded good to excellent reliability for all four assessed muscles: anterior tibialis (ICC = 0.98), longus peroneus (ICC = 0.93), medial gastrocnemius (ICC = 0.71) and lateral gastrocnemius (ICC = 0.98). For stiffness the reliability was excellent (anterior tibialis (ICC = 0.98), longus peroneus (ICC = 0.98), medial gastrocnemius (ICC = 0.92) and lateral gastrocnemius (ICC = 0.98)). For decrement (characterizing the elasticity) we recorded good to excellent the intra-rater reliability (anterior tibialis (ICC = 0.95), longus peroneus (ICC = 0.74), medial gastrocnemius (ICC = 0.82) and lateral gastrocnemius (ICC = 0.87). Handheld dynamometry displayed excellent isometric muscle force measurement reliability of both ankle dorsiflexors (ICC = 0.98) and ankle plantar flexors (ICC = 0.97).

## 4. Discussion

Our study aimed to assess the biomechanical and viscoelastic intrinsic properties of ankle muscles, as well as muscle force in a specific patient category. The patients who have undergone a surgical procedure after a pilon fracture are not so frequently addressed in research in comparison to those with ankle fractures or ankle sprains. As function in patients after a pilon fracture is impaired, with deficits in ankle joint range of motion, altered gait biomechanics, and reduced muscle strength [1,10], we envisaged that muscle assessment could provide data to improve rehabilitation outcomes.

In our study, the gender distribution was in favor of men (17 men vs. 5 women). This is in accordance with general statistics data. The tibial pilon fractures are more common in men than women by a ratio of 3:1 [21].

Potential confounding factors, including pain inhibition, residual edema, duration of immobilization, and time from surgery to testing, were accounted for. All patients were evaluated 12–24 months after surgery, by which time pain and swelling had subsided and full weight-bearing had been reestablished, reducing the influence of early postoperative changes.

The intrinsic properties of peri-articular ankle muscles—including their viscoelasticity, passive stiffness, elasticity, and the behavior of the non-contractile components—are critical for everyday activities such as gait, climbing stairs, and other weight-bearing activities [4,5,22]. These properties are determined by the extracellular matrix composition—the amount, type, and cross-linking of collagen; the abundance, size, and sulfation pattern of proteoglycans; the content of glycosaminoglycans; and the hydration status (water content) of the tissue [6,23]. After fracture and subsequent surgery, scar formation and fibrosis may alter these components: there can be excessive deposition of particular collagen types (e.g., type I vs. type III), abnormal crosslinking (both enzymatic and non-enzymatic, including advanced glycation end products), altered proteoglycan composition (both qualitative and quantitative changes), and disturbed water balance in the extracellular matrix [6,24,25]. These changes can result in increased stiffness, decreased flexibility, poor stress relaxation and creep, limited range of motion, and ultimately poor functional recovery unless rehabilitation directly addresses them (e.g., through mechanical loading, stretching, or other modalities that affect remodeling).

In the context of pilon fractures, meta-analytic data indicate that external fixation and ORIF differ in their soft-tissue complication rates, possibly due to the degree of dissection, exposure, and local vascular compromise [26]. Also, staging ORIF with temporary external fixation allows soft tissues to recover before large incisions are made and fixation hardware is implanted, thereby reducing soft-tissue injury and resulting scarring [3]. These factors may help explain the altered biomechanical properties (e.g., increased stiffness, reduced elasticity) we observed in the muscles of the affected side.

The study by Stefaniak W et al. on male athletes with chronic ankle instability showed excellent test–retest reliability (intraclass correlation coefficients ranging from 0.958 to 0.999) for all myotonometry measurements of the anterior tibialis, peroneus longus, medial gastrocnemius, and lateral gastrocnemius muscles [12].

Our results showed a significantly higher frequency (the intrinsic tension or tone of tissue at rest) in the medial and lateral gastrocnemius (2.57 Hz and 2.09 Hz, respectively) on the affected side in patients after a pilon fracture, compared with the dominant side in healthy controls. The study of Serra-Añó et al. [14] aimed to explore the impact of a previous history of lateral ankle sprain. Their results showed a significantly increased frequency in the lateral gastrocnemius (mean difference of 1.25 Hz) in men without pain-related symptoms and with a previous history of lateral ankle sprain.

Stiffness is a biomechanical property that reflects the resistance of a muscle to an external force that deforms its initial shape [13]. In our study we recorded higher values in patients than in controls. The differences were statistically significant in the medial and lateral gastrocnemius muscle for both the affected and non-affected sides. Although we did not find any significant differences for anterior tibialis and longus peroneus, the values were relatively similar to the study of Serra-Añó P et al. [14] performed on males with a history of lateral ankle sprain (affected side: anterior tibialis 460.9 ± 145.7 N/m vs. 496.77 ± 83.51 N/m; longus peroneus 438.1 ± 133.2 N/m vs. 439.15 ± 76.41 N/m).

Logarithmic decrement defines elasticity. This is a biomechanical property of soft tissues that characterizes the ability to return to its original shape after deformation. In our study, we noted statistically significantly lower values in patients than in healthy subjects for the medial gastrocnemius muscle on both the affected and non-affected sides. However, there were no significant differences in elasticity for the anterior tibialis and the longus peroneus. The research of Stefaniak W. et al. [12] on male athletes with chronic ankle instability, also found no significant difference in elasticity in comparison to healthy male athletes.

The two viscoelastic properties assessed by myotonometry were mechanical stress relaxation time and creep. Creep is the property of progressive deformation under constant stress, reflecting the tissue’s viscosity [27,28].

In our study, we observed that patients presented a significantly lower stress relaxation time in the medial and lateral gastrocnemius for both affected and non-affected sides in comparison to controls (medial gastrocnemius: 17.68 ms vs. 20.65 ms, and 16.6 ms vs. 19.51 ms, respectively; lateral gastrocnemius: 15.44 ms vs. 20.16 ms, and 14.07 ms vs. 19.61 ms). The study of Stefaniak W et al. [12] also showed a decreased relaxation time of medial and lateral gastrocnemius (18.6 ms and 18 ms, respectively, in male athletes with chronic ankle instability vs. 19.7 ms and 19.4 ms, respectively, in healthy athletes). Moreover, patients with surgically treated pilon fractures were characterized by significantly lower values of creep in the medial and lateral gastrocnemius muscles for both affected and non-affected sides. Jansen et al., analyzing changes in gait pattern after pilon fractures, noted that dynamic pedography showed a disturbed walking pattern. Under the fourth and fifth metatarsal regions, there was higher loading on the healthy side compared with the injured limb (fourth metatarsal: 148 ± 75 N vs. 126 ± 43 N, *p* < 0.05; fifth metatarsal: 62 ± 39 N vs. 49 ± 29 N, *p* < 0.05) [29]. In our study, greater loading on the non-surgical fourth and fifth metatarsal regions may be an explanation for the myotonometric changes in the left non-injured lateral gastrocnemius muscle (increased stiffness, decreased stress relaxation time, and reduced ratio of relaxation time to deformation time).

The use of a handheld dynamometer, such as the MicroFET2, represents a practical and reliable method for assessing isometric muscle strength in both clinical and research settings. Previous work by Mentiplay et al. [16] demonstrated that handheld dynamometry provides good to excellent intra- and inter-rater reliability when compared with gold-standard isokinetic devices, with intraclass correlation coefficients exceeding 0.70 across multiple lower-limb muscle groups. Similarly, Roxburgh et al. [17] confirmed excellent relative reliability of the MicroFET2 in patients with severe lower-limb osteoarthritis, reporting intraclass correlation coefficients of 0.92–0.99. These results support the validity of our method and suggest that the strength differences observed between patients with pilon fractures and healthy controls are unlikely to be due to measurement error. In line with the reliability of handheld dynamometry, our results pointed out significant deficits in isometric strength of both ankle dorsiflexors and plantar flexors on the affected side in patients with surgically treated pilon fractures, compared with the dominant side of healthy controls. These reductions are in line with the expected consequences of immobilization, disuse atrophy, and altered neuromuscular recruitment following high-energy ankle trauma and surgery. The absence of significant differences between groups on the contralateral unaffected side indicates that the strength deficits observed are not attributable to systemic factors such as age or general deconditioning.

The deficits in both dorsiflexors and plantar flexors of the affected side are clinically relevant, as they may impair ankle stability, gait propulsion, and the ability to perform functional tasks such as stair climbing or rising from a seated position. Rehabilitation programs should focus on gradually strengthening these muscle groups and restoring mobility to enhance functional recovery and reduce long-term disabilities after pilon fractures. The rehabilitation program will include exercises for ankle range of motion (dorsiflexion, plantar flexion, inversion, and eversion) and strengthening (concentric contractions of the anterior, posterior, and peroneus muscle groups). The single-leg and stair-climbing and descending exercises will be added to improve balance and ankle stability. To our knowledge, no studies have been published on the assessment of myotonometric properties of the ankle muscles in patients with unilateral pilon fractures. The assembly of data on the biomechanical and viscoelastic properties and muscle strength of ankle muscles is a target of our study to develop a tailored exercise program for a particular patient category.

### 4.1. Future Directions

Future longitudinal studies are needed to monitor the evolution of muscle biomechanical and viscoelastic properties at different stages of recovery, as these characteristics may change over time. The patients who had suffered a tibial pilon fracture will benefit from the findings of the current study as the rehabilitation programs will also address the biomechanical and viscoelastic properties, as well as the reduced isometric force of the ankle muscles.

### 4.2. Limitations

The small sample size, single-center design, previous injuries and gender differences represent limitations of the current study. Although a similar surgical approach was used across cases, differences in healing potential and patient compliance may have influenced outcomes. Another limitation is that postoperative rehabilitation was not standardized; each patient followed an individualized program based on their access to physiotherapy and compliance, potentially introducing variability in muscle recovery. However, despite these limitations, we were able to avoid bias and obtain a more accurate analysis of myotonometric parameters by implementing selective inclusion and exclusion criteria.

## 5. Conclusions

Patients with surgically treated unilateral tibial pilon fractures showed reduced isometric muscle strength in ankle dorsiflexors and plantar flexors on both the affected and unaffected limbs, compared to healthy controls. Myotonometry showed increased frequency and stiffness, along with decreased viscoelastic parameters (stress relaxation time and creep) in the medial and lateral gastrocnemius muscles on both sides.

## Figures and Tables

**Table 1:** Characteristics of patients and controls.

	Patients (*n* = 22)	Controls (*n* = 22)	*p*
Age (years), mean (SD)	44 (10.1)	43.14 (7.7)	0.75
Gender			
Males (*n*)	17	17	
Females (*n*)	5	5	
Height (cm), mean (SD)	178.4 (7.01)	174.9 (8.7)	0.65
Weight (kg), mean (SD)	95.5 (19.25)	87.6 (10.3)	0.055
BMI (kg/m^2^), mean (SD)	31.51 (5.54)	28.9 (6.9)	0.07

SD: standard deviation; *n*: number of subjects; BMI: body mass index.

**Table 2:** Ankle range of motion.

	Patients (*n* = 22)		Controls (*n* = 22)		*p* ^a^	*p* ^b^
	Right Side	Left Side	Right Side	Left Side		
Dorsiflexion (°) (mean ± SD)	6.8 ± 1.9	18.3 ± 5.9	22.1 ± 4.0	22.4 ± 4.1	**<0.0001**	0.15
Plantar flexion (°) (mean ± SD)	24.3 ± 13.2	39.5 ± 5.3	44.8 ± 2.5	44.1 ± 2.7	**<0.0001**	0.055
Inversion (°) (mean ± SD)	17.2 ± 11.0	26.8 ± 7.7	32.9 ± 6.4	33.4 ± 7.0	**<0.0001**	0.06
Eversion (°) (mean ± SD)	9.1 ± 8.5	18.7 ± 9.6	23.3 ± 9.0	23.1 ± 8.7	**<0.0001**	0.8

SD: standard deviation; *n*: number of subjects; *p* ^a^: comparison of right ankle range of motion of patients and controls; *p* ^b^: comparison of left ankle range of motion of patients and controls; bold values: *p* < 0.05 indicates statistical significance.

**Table 3:** The isometric muscle force in patients and controls.

	Patients (*n* = 22)		Controls (*n* = 22)		*p* ^a^	*p* ^b^
	Right Side	Left Side	Right Side	Left Side		
Ankle dorsiflexors (N) (mean ± SD)	137.1 ± 28.82	174.3 ± 28.21	180.5 ± 45.85	166.9 ± 49.26	**0.0005**	0.52
Ankle plantar flexors (N) (mean ± SD)	171.8 ± 36.44	201.7 ± 35.25	233.3 ± 70.83	210 ± 75.48	**0.0008**	0.64

N: newton; SD: standard deviation; *n*: number of subjects; *p* ^a^: comparison of right ankle muscle force of patients and controls; *p* ^b^: comparison of left ankle muscle force of patients and controls; bold values: *p* < 0.05 indicates statistical significance.

**Table 4:** Myotonometric parameters of the anterior tibialis muscle in patients and controls.

	Patients (*n* = 22)		Controls (*n* = 22)		*p* ^a^	*p* ^b^
	Right Side	Left Side	Right Side	Left Side		
Frequency (Hz) (mean ± SD)	20.80 ± 4.33	21.49 ± 4.39	20.22 ± 4.7	19.98 ± 2.74	0.66	0.17
Stiffness (N/m) (mean ± SD)	460.9 ± 145.7	481± 143.4	448.5 ± 163.5	423.4 ± 66.3	0.79	0.09
Decrement (mean ± SD)	0.97 ± 0.17	0.96 ± 0.15	0.94 ± 0.21	0.91 ± 0.13	0.68	0.28
Relaxation (ms) (mean ± SD)	13.21 ± 3.47	12.73 ± 3.41	13.57 ± 3.6	13.47 ± 2.24	0.73	0.39
Creep (mean ± SD)	0.85 ± 0.19	0.82 ± 0.17	0.87 ± 0.21	0.85 ± 0.13	0.79	0.50

SD: standard deviation; *n*: number of subjects; *p* ^a^: comparison of right anterior tibialis myotonometric parameters of patients and controls; *p* ^b^: comparison of left anterior tibialis myotonometric parameters of patients and controls.

**Table 5:** Myotonometric parameters of the longus peroneus muscle in patients and controls.

	Patients (*n* = 22)		Controls (*n* = 22)		*p* ^a^	*p* ^b^
	Right Side	Left Side	Right Side	Left Side		
Frequency (Hz) (mean ± SD)	19.43 ± 4.19	19.81 ± 4.04	18.65 ± 4.46	19.05 ± 4.1	0.55	0.54
Stiffness (N/m) (mean ± SD)	438.1 ± 133.2	428.2 ± 115	388.2 ± 107	398.3 ± 97.6	0.17	0.37
Decrement (mean ± SD)	0.98 ± 0.22	0.90 ± 0.21	1.02 ± 0.28	0.94 ± 0.16	0.57	0.56
Relaxation (ms) (mean ± SD)	13.47 ± 3.09	13.54 ± 3.29	14.58 ± 3.77	14.45 ± 3.59	0.30	0.39
Creep (mean ± SD)	0.87 ± 0.15	0.85 ± 0.18	0.91 ± 0.22	0.91 ± 0.21	0.45	0.37

SD: standard deviation; *n*: number of subjects; *p* ^a^: comparison of right longus peroneus myotonometric parameters of patients and controls; *p* ^b^: comparison of left longus peroneus myotonometric parameters of patients and controls.

**Table 6:** Myotonometric parameters of the medial gastrocnemius muscle in patients and controls.

	Patients (*n* = 22)		Controls (*n* = 22)		*p* ^a^	*p* ^b^
	Right Side	Left Side	Right Side	Left Side		
Frequency (Hz) (mean ± SD)	16.17 ± 2.73	16.64 ± 2.23	13.6 ± 2.22	14.15 ± 1.98	**0.0014**	**0.0003**
Stiffness (N/m) (mean ± SD)	308 ± 73.81	331.8 ± 64.11	252.9 ± 51.64	264.7 ± 43.57	**0.0063**	**0.0002**
Decrement (mean ± SD)	1.16 ± 0.16	1.14 ± 0.14	1.35 ± 0.16	1.34 ± 0.24	**0.0003**	**0.002**
Relaxation (ms) (mean ± SD)	17.68 ± 3.63	16.6 ± 3.35	20.65 ± 3.87	19.51 ± 3.23	**0.012**	**0.0064**
Creep (mean ± SD)	1.09 ± 0.21	1.04 ± 0.2	1.27 ± 0.23	1.2 ± 0.19	**0.011**	**0.0095**

SD: standard deviation; *n*: number of subjects; *p* ^a^: comparison of right medial gastrocnemius myotonometric parameters of patients and controls; *p* ^b^: comparison of left medial gastrocnemius myotonometric parameters of patients and controls; bold values: *p* < 0.05 indicates statistical significance.

**Table 7:** Myotonometric parameters of the lateral gastrocnemius muscle in patients and controls.

	Patients (*n* = 22)		Controls (*n* = 22)		*p* ^a^	*p* ^b^
	Right Side	Left Side	Right Side	Left Side		
Frequency (Hz) (mean ± SD)	17.73 ± 2.47	19 ± 3.23	14.64 ± 3.26	15.48 ± 4.6	**0.001**	**0.005**
Stiffness (N/m) (mean ± SD)	359.4 ± 53.12	416 ± 104.9	270.4 ± 66.98	289.5 ± 85.42	**<0.0001**	**0.012**
Decrement (mean ± SD)	1.24 ± 0.21	1.23 ± 0.22	1.32 ± 0.21	1.49 ± 0.39	0.19	**0.013**
Relaxation (ms) (mean ± SD)	15.44 ± 2.35	14.07 ± 3.26	20.16 ± 4.22	19.61 ± 4.74	**<0.0001**	**<0.0001**
Creep (mean ± SD)	0.97 ± 0.15	0.89 ± 0.2	1.25 ± 0.25	1.22 ± 0.28	**<0.0001**	**<0.0001**

SD: standard deviation; *n*: number of subjects; *p* ^a^: comparison of right lateral gastrocnemius myotonometric parameters of the patients and controls; *p* ^b^: comparison of left lateral gastrocnemius myotonometric parameters of the patients and controls; bold values: *p* < 0.05 indicates statistical significance.

## Data Availability

The data presented in this study are available upon request from the corresponding author (E.C.A.).

## Abbreviations

The following abbreviations are used in this manuscript:

ORIF	Open Reduction and Internal Fixation
ROM	Range Of Motion
CAI	Chronic Ankle Instability
SD	Standard Deviation
